# Zoonotic *Chlamydiaceae* Species Associated with Trachoma, Nepal

**DOI:** 10.3201/eid1912.130656

**Published:** 2013-12

**Authors:** Deborah Dean, James Rothschild, Anke Ruettger, Ram Prasad Kandel, Konrad Sachse

**Affiliations:** Children's Hospital Oakland Research Institute, Oakland, California, USA (D. Dean, J. Rothschild);; University of California, San Francisco, California, USA (D. Dean);; University of California, Berkeley, California, USA (D. Dean);; Friedrich-Loeffler-Institut, Jena, Germany (A. Ruettger, K. Sachse);; Lumini Eye Hospital, Bhairahawa, Nepal (R.P. Kandel)

**Keywords:** Chlamydiaceae, zoonotic transmission, microarray, species and strain typing, zoonoses, Nepal, trachoma, bacteria

## Abstract

Trachoma is the leading cause of preventable blindness. Commercial assays do not discriminate among all *Chlamydiaceae* species that might be involved in trachoma. We investigated whether a commercial Micro-ArrayTube could discriminate *Chlamydiaceae* species in DNA extracted directly from conjunctival samples from 101 trachoma patients in Nepal. To evaluate organism viability, we extracted RNA, reverse transcribed it, and subjected it to quantitative real-time PCR. We found that 71 (70.3%) villagers were infected. ArrayTube sensitivity was 91.7% and specificity was 100% compared with that of real-time PCR. Concordance between genotypes detected by microarray and *ompA* genotyping was 100%. Species distribution included 54 (76%) single infections with *Chlamydia trachomatis*, *C. psittaci*, *C. suis,* or *C. pecorum,* and 17 (24%) mixed infections that includied *C. pneumoniae.* Ocular infections were caused by 5 *Chlamydiaceae* species. Additional studies of trachoma pathogenesis involving *Chlamydiaceae* species other than *C. trachomatis* and their zoonotic origins are needed.

Trachoma was first recognized as an ocular disease in the 27th century BC in China (*1*). Subsequent reports documented the disease among the Egyptians and Greeks in the 19th and 1st centuries BC, respectively. The word trachoma derives from the Greek word for rough swelling, referring to the follicles that appear on the tarsal conjunctiva. Epidemic trachoma was spread from the Middle East to Europe during the Crusades and was a major cause of blindness during the Napoleonic era (*1*). The disease was eliminated from most industrialized countries after the industrial revolution, which heralded the institution of improved sanitation, hygiene, and nutrition. Currently, trachoma prevalence is hypoendemic, mesoendemic, and hyperendemic among populations residing in tropical developing countries.

During the past few decades, rates of trachoma have increased; in response, at the end of the 1990s, the World Health Organization developed the SAFE program with the goal of eliminating blinding trachoma by the year 2020. SAFE refers to Surgery, Antibiotics, Facial cleanliness, and Environmental improvements, specifically, surgery to correct trichiasis (in-turned eyelashes), oral antimicrobial drugs to treat *Chlamydia trachomatis* infections, facial cleanliness to decrease ocular infections, and environmental improvements such as latrines and wells to provide clean water. Unfortunately, most efforts have focused on the surgery and antimicrobial drug components and had disappointing results. Trichiasis often recurs months to years after surgery for 25%–75% of patients (*2*,*3*) and can be a result of reinfection (*3*). Infection often returns to pretreatment levels 6–24 months after termination of treatment (*4*,*5*). The recurrence of infection and disease is probably multifactorial. There is evidence that oral treatment of *C. trachomatis* infection blunts the immune response, increasing the patient’s susceptibility to reinfection (*4*). Furthermore, additional species of *Chlamydiaceae*, namely *Chlamydia pneumoniae* and *C. psittaci*, have been implicated in trachomatous disease by our group (*6*) and by another independent research group from Paris working in Guinea, Africa (*7*). To eliminate infections with species other than *C. trachomatis,* longer treatment intervals might be required (*8*).

Although some *Chlamydiaceae* screening tests and strain-typing methods exist, they are expensive, are time-consuming, require trained personnel, and are available only in specialized laboratories; most do not discriminate among species of *Chlamydiaceae*. The tests or methods include serotyping of the major outer membrane protein by using monoclonal or polyclonal antibodies that are species or genus specific; commercial nucleic acid amplification tests for *C. trachomatis* only (*9*); conventional species-specific and genus-specific PCRs (*10*); direct sequence analysis of *ompA*, 16S rRNS, or 23S rRNA genes (*11*,*12*); multilocus sequence typing for *C. trachomatis (**13**, **14**)* and other species (*14*); real-time (RT)-PCR (*6*,*15*), multilocus variable number tandem repeat analysis (*16*); and the commercial micro ArrayTube or ArrayStrip (Alere Technologies, Jena, Germany) (*17*). Serotyping requires a cultured isolate, and techniques that involve sequencing might not be able to detect mixed-strain or mixed-species infections unless multiple strain–specific or species-specific primers are used, which require sufficient quantities of DNA. The advantage of the ArrayTube or ArrayStrip is that minimal DNA is required for amplification, and the hybridization patterns indicate species-specific nucleotide polymorphisms in regions of high sequence similarity.

The commercial ArrayTube assay has been successfully used to identify mixed infections among animals infected with multiple species of *Chlamydiaceae (**18**,**19**)*. Because of these benefits, we investigated whether the ArrayTube could discriminate among *Chlamydiaceae* species in DNA that was extracted directly from conjunctival samples from trachoma patients residing in a trachoma-endemic region of Nepal. We also evaluated the correlation of the ArrayTube test with *ompA* genotypes. As an independent test for viability of *Chlamydiaceae* organisms, RNA was isolated from the same samples and tested by quantitative RT-PCR (qRT-PCR).

## Methods

### Study Population and Samples

We used a table of random numbers to randomly select 101 villagers, 1–65 years of age, who had follicular trachomatous inflammation and/or intense trachomatous inflammation and who resided in a trachoma-endemic region of the Lumbini Zone of southwestern Nepal. Patients were enrolled after they provided informed consent. For trachoma grading, we used the modified World Health Organization scale. Upper tarsal conjunctival samples were obtained by using dacron swabs (Hycor Biomedical, Portland, ME, USA), which were immediately placed in M4 transport media (Remel, Lenexa, KS, USA) and stored in liquid nitrogen as described (*6*). To avoid contamination, study personnel changed gloves between participants.

### Ethics Statement

 The study was approved by institutional review boards of the Nepal Netra Jhoti Shang (Kathmandu, Nepal) and the Children’s Hospital Oakland Research Institute (Oakland, CA, USA). Informed consent was obtained from each study participant. Oral consent was approved by both institutional review boards because of the high rate of illiteracy among the population. Consent was documented on the form by the team member who obtained the consent; the team member obtaining the consent signed the form stating that consent had been obtained. Since some study participants were minors, parents provided consent for their child to participate.

### RNA and DNA Purification 

 Genomic DNA was extracted from the conjunctival swab samples by using the Roche High Pure kit (Roche, Pleasanton, CA, USA), and RNA was extracted by using the RNeasy kit (QIAGEN, Valencia, CA, USA) as described (*6*,*20*). RNA was reverse transcribed to cDNA by using a TaqMan reverse transcription kit (Applied Biosystems, Foster City, CA, USA) as described (*6*,*20*). DNA and RNA were stored at −80°C until use.

### DNA Microarray Assay

 To examine samples for the presence of any of the 9 *Chlamydiaceae* species and *Waddlia chondrophila* and *Simkania negevensis*, we performed the ArrayTube assay as described (*17*,*19*). Briefly, DNA from each sample was amplified and biotin labeled in 40 cycles of 94°C for 30 s, 55°C for 30 s, and 72°C for 30 s by using primers U23F-19 and 23R-22 (Table 1). Hybridization was conducted in the ArrayTube vessel at 58°C for 1 h. After 3 wash steps, hybridization signals were visualized by using streptavidin-conjugated peroxidase-catalyzed precipitation. The resulting patterns were processed by using the ATR-01 ArrayTube reader (Alere Technologies) and the Iconoclust 2.3 program (Alere).

**Table 1:** Oligonucleotide primers used for the ArrayTube, quantitative real-time –PCR, and PCR for subsequent sequencing

Gene	Primers	Primer sequence, 5′→ 3′	Gene location	Base pair	Reference
23S rRNA*	U23F-19	ATTGAMAGGCGAWGAAGGA			(*17*)
	23R-22	biotin-GCYTACTAAGATGTTTCAGTTC			
*Chlamydiaceae* 16S rRNA†	16SrRNA-9	GCGAAGGCGCTTTTCTAATTTAT	734–756‡	76	(*6*)
	16SrRNA-10	CCAGGGTATCTAATCCTGTTTGCT	809–786‡		
*Chlamydia trachomatis ompA*†	OmpA-9	TGCCGCTTTGAGTTCTGCTT	33–52§	75	(*6*)
	OmpA-10	GTCGATCATAAGGCTTGGTTCAG	108–86§		
*C. pneumoniae ompA*†	Cpn ompAF1	ATAGACCTAACCCGGCCTACAATAAG	301–330	108	(*6*)
	Cpn ompAR1	GTGAACCACTCTGCATCGTGTAA	353–333	53	
*C. psittaci* *ompA*†	CpsF	GCAACTCCTACGGAGTCTTAA	260–279	93	(*6*)
	CpsB	GGCATCTTGTAAATGTTTCCCTAT	331–354		
*C. pecorum* *ompA*†	Cp-F	GTTTTCGACAGAGTCCTCAA	208–227	118	This study
	CpRT-R	ATTCTAATTTGCTCTTCTGG	325–305		
*C. abortus* *ompA*†	CpaOMP1-F	GCAACTGACACTAAGTCGGCTACA	763–786	82	(*15*)
	CpaOMP1-R	ACAAGCATGTTCAATCGATAAGAGA	845–821		
*C. suis* *ompA*†	Cs-F	GGAGATTATGTTTTCGATCGC	195–216	122	This study
	Cs-R	TAAGCTGCATTACTCGTTGTTTCA	338–292		
β-actin†	β-actin-3	GGTGCATCTCTGCCTTACAGATC	412–434¶	73	(*6*)
	β-actin-4	ACAGCCTGGATAGCAACGTACAT	52–30#		
C. trachomatis ompA**	ompAF-1	GTGCCGCCAGAAAAAGAT	−60–40§	1542	(*6*)
	ompAR-2	CCAGAAACACGGATAGTGTTATTA	55–31††		
C. pneumoniae ompA**	CPF1	TTACAAGCCTTGCCTGTAGGGA	70–91‡‡	1098	(*8*)
	CPB4	AGAATCTGGACTGACCAGATACGTGAG	1169–1142‡‡		
*C. psittaci* ompA**	Cps-1	GTATTAAAAGTTGATGTGAATAA	217–239§§	1022	(*8*)
	Cps-B4	TTGATTAAGCGTGCTTCACCAGTGATT	1169–1143§§		
*C. suis* ompA**	Cs-F	GGAGATTATGTTTTCGATCGC	195–216	959	This study
	Cs-R	TAGAATCTGAATTGAGCGTTTACGTGA	1154–1128		
*C. pecorum* ompA**	Cp-F	GTTTTCGACAGAGTCCTCAA	208–227	966	This study
	Cp-R	GAATCTGAACTGACCAGATACGTGAG	1173–1148		
16Sr RNA**	16SrRNA-F	CAGTCGAGAATCTTTCGCAAT	362–382c	904	(*6*)
	16SrRNA-R	TACTGCCCATTGTAGCACGTGTGT	1265–1232c		


*Primers used in ArrayTube (Alere Technologies, Jena, Germany). †Primer pairs used for real-time PCR of *ompA* DNA and of cDNA from RNA for *16SrRNA* for detecting *Chlamydiaceae.* ‡Primer location based on reference strain L_2_/434 *16SrRNA* sequence. §Primer location based on reference strain L_2_/434 *ompA* sequence. ¶Primer location based on position within intron 3 of the human β*-actin* sequence. #Primer location based on position within exon 3 of the human β*-actin* sequence. **Primer pairs used for PCR. ‡‡Primer location based on intergenic region of reference strain L_2_/434 downstream of *ompA* sequence. ‡‡Primer location based on *C. pneumoniae* strain TW183 *ompA.* §§Primer location based on *C. psittaci* avian type C strain *ompA.*

### Genus-Specific and Species-Specific qRT-PCR and *ompA* Genotyping

 qRT-PCR was conducted by using genus-specific and species-specific primers (Table 1) along with appropriate controls including β-actin as described (*6*,*20*). Briefly, each reaction contained 1X SYBR Green PCR MasterMix (Applied Biosystems), 300 nmol/L of each primer, and 5 μL of sample DNA in a volume of 25 μL in duplicate in a 96-well plate. The thermocycling consisted of 10 min at 94°C followed by 40 cycles of 15 s at 94°C and 1 min at 60°C. Samples that were positive by qRT-PCR were subjected to PCR with 16S rRNA genus-specific primers in addition to species-specific *ompA* primers (Table 1). The PCR reagents, controls, thermocycling, and sequencing by BigDye Terminator (Applied Biosystems) automated capillary sequencing were used or performed as described (*6*,*20*). In addition, *ompA* genotyping of samples showing monoinfection with *C. trachomatis* was conducted by using the ArrayStrip microarray assay as described (*21*).

### Data Analysis

Outcome variables included single or mixed infection, *Chlamydiaceae* species causing the infection, and *ompA* genotype. The association between discrete variables was analyzed by using the Fisher exact test or the Pearson χ^2^ test by Stata 10 (College Station, TX, USA). A p value of <0.05 was used as the cutoff for determining statistical significance.

## Results

The distribution of villagers by age and sex is shown in Table 2; none lived in the same household. The rate of mixed infections was significantly higher for female than for male participants (p = 0.0011), although the overall rates of infection did not differ. Single or mixed infections did not differ by age group, although the rate of single and mixed infections among those >10 years of age was significantly higher (p = 0.0472). The mean age was 26 years for female and 28 years for male participants.

**Table 2:** Correlation of sex and age with single and mixed *Chlamydiaceae* species infections

Patient variable	Total infected, no. (%); n = 71	p value	Single infections, no. (%); n = 54	p value	Mixed infections, no. (%); n = 17	p value
Sex						
M (n = 33)	22 (66.7)	0.6448	21 (63.6)	0.2025	1 (3.0)	0.0098
F (n = 68)	49 (75.1)		33 (48.5)		16 (23.5)	
Age, y						
1–10 (n = 44)	26 (59.1)	0.0472	19 (43.2)	0.0748	7 (15.9)	1.000
>10 (n = 57)	45 (78.9)		35 (61.4)		10 (17.5)	


For purposes of cross-comparison, the ArrayTube and qRT-PCR analyses were run independently. For the 101 samples, the ArrayTube had a sensitivity of 91.7% and a specificity of 100% compared with sensitivity and specificity of qRT-PCR (Technical Appendix Table). None of the samples yielded positive results for *C. abortus* by ArrayTube or qRT-PCR. Six samples were not detected by the ArrayTube but were *C. trachomatis* positive by qRT-PCR. All samples positive by qRT-PCR were genotyped; the *ompA* genotypes matched 100% of those identified by ArrayTube.

**Figure 1:**
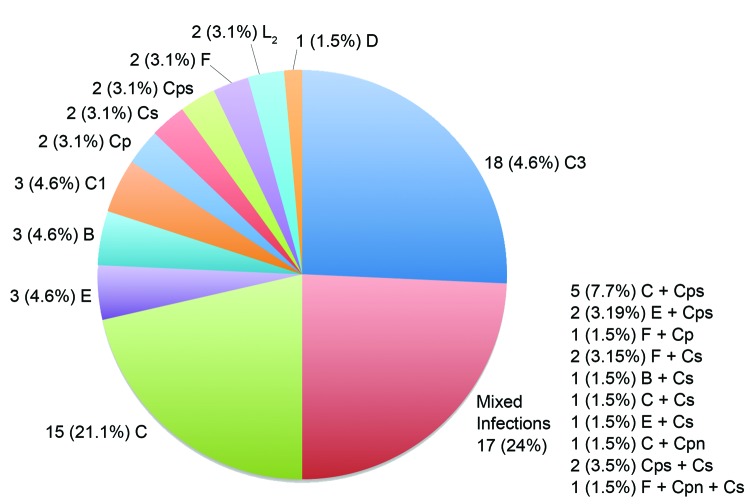
*Chlamydiaceae* infections among 101 villagers residing in a trachoma-endemic region of southwestern Nepal identified by the ArrayTube (Alere Technologies, Jena, Germany), real-time PCR, and *ompA* genotyping. The number and percentage for each infection are shown. Single infections included each species and the designated *ompA* genotypes (n = 71). *C. trachomatis* (Ct) trachoma strain C predominated, but single infections with *C. psittaci* (Cps), *C. pecorum* (Cp), and *C. suis* (Cs) also occurred). Mixed infections included those with Ct, Cps, *C. pneumoniae* (Cpn), Cp, and Cs.

**Figure 2:**
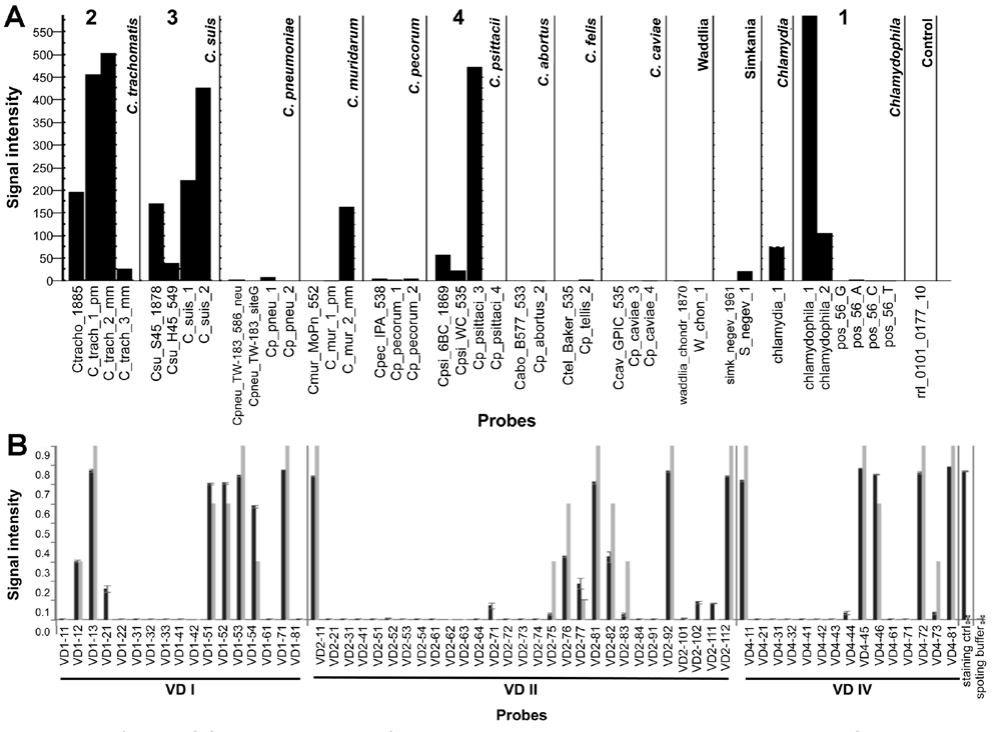
Identification of *Chlamydiaceae* triple infection by using the ArrayTube (Alere Technologies, Jena, Germany) assay. A) Biotinylated PCR product from a DNA extract was hybridized to a DNA microarray carrying species-specific probes from the 23S rRNA gene locus (*17*). Bar graph shows specific hybridization signals for genus *Chlamydia* (1), *C. trachomatis* (2), *C. suis* (3), and *C. psittaci* (4) in sample 67. Other signals represent nonspecific cross-hybridization. B) *omp*A genotyping of the *C. trachomatis* strain from sample 64 conducted by using the ArrayStrip platform that is specific for *C. trachomatis*. The best match of this sample was genotype C. The genotype has been determined by automatic comparison of experimentally obtained (black bars) and theoretically constructed (gray bars) hybridization patterns with use of the software's PatternMatch algorithm. The numerical values of matching score MS (measure of similarity between sample and reference strain) and Delta MS (numerical difference between best and second best match) indicate that the identification is highly accurate (*21*). The rightmost bars represent internal staining control (biotinylated oligonucleotide probe) and spotting buffer (background).

Of the 101 participating villagers, 71 (70.3%) were infected (Figure 1); 26 (37%) of the infections involved a single or mixed infection with a species other than *C. trachomatis* or in combination with *C. trachomatis*. The 54 (76%) single infections were 48 (88.9%) *C. trachomatis,* 2 (3.7%) *C. psittaci*, 2 (3.7%) *C. suis,* and 2 (3.7%) *C. pecorum.* Most infections were caused by *C. trachomatis* strains C, C1, and C3, although urogenital strains B, D, E, F, and L_2_ were also represented. The 17 (24%) mixed infections were 15 (88.2%) *C. trachomatis* plus another species and 2 (11.8%) other species. Specifically, they were 7 (41.2%) *C. trachomatis* plus *C. psittaci*, 5 (29.4%) *C. trachomatis* plus *C. suis*, 2 (11.7%) *C. psittaci* plus *C. suis*, 1 (5.9%) *C. trachomatis* plus *C. pneumoniae*, 1 (5.9%) *C. trachomatis* plus *C. pecorum*, and 1 (5.9%) *C. psittaci* plus *C. suis* and *C. trachomatis.* There were no statistically significant differences by patient age or sex for infections caused by *C. trachomatis* or other species. Figure 2, panel A, shows the ArrayTube assay for the sample from the patient infected with the latter 3 species. Figure 2, panel B, shows an example of microarray-based *omp*A genotyping. 

## Discussion

Although *C. trachomatis* is well established as a cause of trachoma (*1*), the high percentage of infections with other species (37%) found in this study suggests a role for these pathogens in trachoma pathogenesis. In addition, the use of 2 independent methods to detect *Chlamydiaceae*, one of which used RNA to demonstrate the presence of viable *Chlamydiaceae* species in trachomatous eyes, lends further support to this theory. Our findings also support 2 previous studies; 1 conducted by our group and 1 conducted by an independent research group in Paris. Our group detected *C. trachomatis*, *C. psittaci*, and *C. pneumoniae* DNA and RNA among Nepali villagers in all age groups (*6*). Just under 60% of the cases were caused by single or mixed infections with species other than *C. trachomatis*. In addition, infection with each species was significantly associated with antibodies to chlamydial heat shock protein 60, a known virulence factor for the organism associated with inflammation and trachomatous scarring. The Paris group conducted their study in a trachoma-endemic region of Guinea, Africa, and detected *C. trachomatis*, *C. psittaci*, and *C. pneumoniae* among children 1–10 years of age by using RT-PCR that targeted a conserved sequence of 16S rRNA (*7*).

These findings are not surprising for several reasons. Historically, swab samples from persons in a trachoma-endemic area were cultured and then serologically typed by using microimmunofluorescence, a technique that requires technical expertise, and only 1 strain each of *C. pneumoniae* and *C. psittaci* was included. However, cross-reactivity among species has been observed *(**22**)*. Immunoreactivity to other species, therefore, might not be considered as evidence for infection with these pathogens. However, in a study in Sudan where trachoma is hyperendemic, immunoreactivity to all 3 species was found among 3% of persons with clinical trachoma *(**23**)*. Their findings suggest either cross-reactivity or infection with these species. Currently, conjunctival swab samples from patients in trachoma-endemic areas are usually only tested by *C. trachomatis–*specific commercial nucleic acid amplification tests or by *C. trachomatis–*specific in-house PCR. Although a pan-Chlamydiales PCR is available, it might not detect mixed infections because an additional cloning step is required *(**24**)*. Furthermore, this test has not been applied to samples from persons in a trachoma-endemic area. Consequently, unless investigators think to use specific tests to look for other species, they will not be detected.

Our study identified ocular infections with 5 *Chlamydiaceae* species among trachoma patients. Over the past 5 years, improved technology has enabled increasing identification of single and mixed infections with *Chlamydiaceae* species among mammals and birds (*10*,*19*,*25*). Co-infections with *C. psittaci* and *C. abortus* have been found in cow milk, and co-infections with *C. abortus* and *C. pecorum* have been identified in conjunctival and nasal swab specimens from calves (*19*). Mixed infections among bovine abortion cases have included *C. abortus* and *C. suis (**25**)*. When cloacal swab samples and fecal samples from pigeons were tested, mixed infections with *C. psittaci,* combined with *C. pecorum*, *C. abortus,* or *C. trachomatis,* along with unclassified *Chlamydiaceae* spp., were discovered (*18*). Most studies used the commercial ArrayTube or ArrayStrip.

Prevalence of ocular infections with *Chlamydiaceae* species in different mammals is high, which supports the notion that humans also are probably susceptible to ocular infection and disease with zoonotic species. A recent study identified several *Chlamydiaceae* species infecting the diseased and healthy eyes of sheep; these species were *C. abortus*, *C. pecorum*, *C. suis*, and uncultured *Chlamydia*-like organisms (*26*). *C. psittaci* has been associated with ocular diseases in sheep and koalas (*27*). *C. suis* is also well known as a cause of conjunctivitis among pigs (*28*), and *C. pecorum* is associated with keratoconjunctivitis among sheep and goats (*26*). One study tracked *Chlamydiaceae* infections among humans and their domesticated animals and found *C. psittaci* on ocular swabs from humans, cattle, buffaloes, sheep, and goats inhabiting the same compounds (*29*).

The plethora of mixed infections, along with single infections with various *Chlamydiaceae* species, among domesticated animals (such as ducks, pigs, cows, sheep, goats, horses, and cattle) suggests multiple opportunities for transmission to humans and development of disease. *C. pneumoniae (**30**)* and *C. psittaci (**31**,**32**)* are known to be transmissible from human to human. Alternatively, infection and reinfection from animals could be another mechanism for transmission in which human-to-human transmission, which is necessary for the human-confined pathogen *C. trachomatis*, would not be required. In addition, clinical outcomes could be worse when caused by mixed chlamydial infections rather than monoinfection, as was suggested in a study of ovine abortion (*33*).

A major question is whether *C. trachomatis* strains that cause sexually transmitted diseases (STDs) or *Chlamydiaceae* species that cause zoonotic infection are capable of causing chronic infection and the cycles of reinfection that are characteristic of trachoma caused by *C. trachomatis*. STD-strain infections among children causing trachoma-like disease have been reported. In a study by Harrison et al. (*34*), urogenital strain J was isolated from the conjunctiva of a Navajo child who had trachoma. This strain was also noted to have been isolated from the urogenital tract of Native American women in the same area. Mordhorst et al. (*35*) isolated strains Ba, D, E, G, and H from 14 patients in Denmark for whom onset of infection and trachoma occurred during childhood. Of these 14 patients, trachomatous disease was severe for 5. We have also documented trachoma-like disease caused by *C. psittaci* and *C. pneumoniae* in the United States (*8*). Another report notes the isolation of *C. pneumoniae* from a laboratory technician with acute follicular conjunctivitis who had been working with the agent (*36*). Incidents of ocular infection with *C. suis* have occurred among pig farm and slaughterhouse workers (D. Vanrompay, pers. comm.); however, these infections were asymptomatic.

Animal models of trachoma also lend support to the pathogenic role of STD strains and other zoonotic *Chlamydiaceae* species in trachoma. STD strains E and G have been shown to produce severe ocular disease similar to trachoma in macaque or baboon models of trachoma (*37*–*39*). There is also a guinea pig model of trachoma in which *C. caviae* has been shown to produce pathologic changes similar to those of trachoma (*40*). These collective findings indicate that multiple zoonotic species can probably infect the eyes of humans and might contribute to trachomatous disease pathogenesis.

In our study, we randomly selected villagers with follicular or intense trachomatous inflammation to screen for *Chlamydiaceae* infections. None of the selected villagers lived in the same household. Most infections were with trachoma C strains, although urogenital strains B, D, E, F, and L_2_ were also detected. We have documented urogenital infections in trachoma patients in Nepal but not with L_2_ (*6*), perhaps because of the small sample size. We identified 4 other zoonotic species as etiologic agents in single or mixed infections. These species are common among domesticated animals (such as pigeons, pigs, cows, and buffaloes), which in trachoma-endemic communities are commonly kept for consumption or agricultural purposes. In our previous study (*6*), we tested for only *C. psittaci* and *C. pneumoniae* in addition to *C. trachomatis*.

The mixed infection rate of 24% found in our study was comparable to the rate of 35% found in our previous study, although the higher rate might have reflected the testing of multiple family members who would have had the same exposure to infected animals (*6*). The significantly higher rate of mixed infections among female participants is consistent with their societal role of caring for domesticated animals and the enhanced opportunity for contact with potentially infected animals. No significant associations were found between age or sex and infecting species. This finding is similar to that of the previous study (*6*), indicating that these species are probably prevalent in the communities and that all villagers are susceptible to zoonotic infection.

The ArrayTube was 91.7% sensitive and 100% specific compared with qRT-PCR. That 5 samples were negative by the ArrayTube might reflect the higher sensitivity of amplifying a single target in qRT-PCR compared with amplifying multiple targets in a single ArrayTube assay. Nonetheless, the ArrayTube assay is a relatively quick assay for screening populations in trachoma-endemic areas for *Chlamydiaceae* species. It is ideally suited for detecting mixed infections that might be missed by tests that target a single species, that amplify only the most abundant species in the sample, or that require additional DNA for multiple strain-specific or species-specific amplifications.

Identifying *Chlamydiaceae* species distribution among persons in a trachoma-endemic area is critical for understanding disease prevalence and instituting appropriate therapeutic regimens for the specific species (*8*). For assessment of the prevalence of infections caused by all *Chlamydiaceae* species and for a better understanding of their zoonotic origins, additional studies using the ArrayTube are warranted in other trachoma-endemic countries worldwide. For prevention of transmission from animal to human hosts, interventions will probably need to be instituted. Our results, then, represent findings that could help guide the World Health Organization program for eliminating blinding trachoma by the year 2020. Finally, understanding the full effects of multiple *Chlamydiaceae* species on the epidemiology, immunopathology, and disease outcome of trachoma will be a major research challenge. Although additional studies are needed, on the basis of our findings, vaccine design will probably need to consider the potential diversity of the host immune response to different *Chlamydiaceae* pathogens.

Technical Appendix Association of ArrayTube results with real-time PCR and *ompA* genotyping.
