# Supplementary material for: Zoonotic Chlamydiaceae Species Associated with Trachoma, Nepal
Source: Emerg Infect Dis. 2013 Dec;19(12):1948–55. doi: 10.3201/eid1912.130656 (PMC3840858; doi:10.3201/eid1912.130656)
Supplement: Technical Appendix — Association of ArrayTube results with real-time PCR and ompA genotyping. [file 13-0656-Techapp-s1.pdf]

Article DOI: http://dx.doi.org/10.3201/eid1912.130656

Zoonotic *Chlamydiaceae* Species associated with Trachoma, Nepal

Technical Appendix

Technical Appendix Table. Association of ArrayTube results with real-time PCR and *ompA* genotyping*

Nepal sample no.	Species identity (by ArrayTube)	*ompA* genotype(s)†
1	*Chlamydia trachomatis*	C
2	*C. trachomatis*	E
3	*C. trachomatis*	L2
4	*C. trachomatis*	L2
5	*C. trachomatis* + *C. suis*	B + *C. suis*
6	*C. trachomatis* + *C. suis*	F + *C. suis*
7	*C. trachomatis*	E
8	*C. trachomatis* + *C. suis*	C + *C. suis*
9	*C. trachomatis*	C
10	*C. trachomatis*	C
11	Negative	Negative
12	*C. trachomatis*	C
13	*C. trachomatis*	C
14	*C. trachomatis*	C
15	*C. trachomatis* + *C. suis*	F + *C. suis*
16	*C. trachomatis*	C
17	*C. trachomatis*	C
18	*C. trachomatis*	C
19	*C. trachomatis*	C
20	Negative	Negative
21	*C. trachomatis*	C
22*	Negative	C
23*	Negative	B
24	*C. trachomatis*	C
25	Negative	Negative
26*	Negative	C
27*	Negative	E
28	Negative	Negative
29	*C. trachomatis*	D
30	Negative	Negative
31	*C. trachomatis*	C
32	*C. psittaci* + *C. suis*	*C. psittaci* + *C. suis*
33	*C. trachomatis* + *C. psittaci*	C + *C. psittaci*
34	*C. trachomatis* + *C. suis*	E + *C. suis*
35	*C. trachomatis*	C
36	*C. trachomatis*	C
37	*C. trachomatis*	C
38	Negative	Negative
39	Negative	Negative
40	*C. trachomatis*	C
41	Negative	Negative
42	*C. trachomatis*	C
43	*C. trachomatis*	C
44	*C. psittaci*	*C. psittaci*
45	Negative	Negative
46	Negative	Negative
47	Negative	Negative
48	*C. suis*	*C. suis*
49	Negative	Negative
50	*C. suis*	*C. suis*
51	Negative	Negative
52	*C. trachomatis* + *C. psittaci*	E + *C. psittaci*
53	*C. trachomatis*	C
54	Negative	Negative

Nepal sample no.	Species identity (by ArrayTube)	*ompA* genotype(s)†
55	Negative	C
56	*C. trachomatis*	C
57	Negative	Negative
58	Negative	Negative
59	Negative	Negative
60	Negative	Negative
61	Negative	Negative
62	Negative	Negative
63*	Negative	F
64	*C. pecorum*	*C. pecorum*
65	Negative	Negative
66	*C. trachomatis*	C
67	*C. trachomatis* + *C. suis* + *C. psittaci*	F + *C. suis* + *C. psittaci*
68	*C. trachomatis*	C
69	*C. trachomatis*	C
70	*C. trachomatis*	C
71	*C. trachomatis*	B
72	*C. trachomatis*	C
73	*C. psittaci*	*C. psittaci*
74	Negative	Negative
75	*C. pecorum*	*C. pecorum*
76	*C. trachomatis*	C
77	Negative	Negative
78	Negative	Negative
79	*C. trachomatis*	C
80	*C. trachomatis*	B
81	Negative	Negative
82	*C. trachomatis*	C
83	*C. trachomatis* + *C. psittaci*	C + *C. psittaci*
84	*C. trachomatis*	C
85	*C. trachomatis*	F
86	*C. trachomatis*	C
87	*C. trachomatis* + *C. pneumoniae*	C + *C. pneumoniae*
88	Negative	Negative
89	Negative	Negative
90	*C. psittaci* + *C. suis*	*C. psittaci* + *C. suis*
91	Negative	Negative
92	*C. trachomatis*	C
93	*C. trachomatis*	C
94	Negative	Negative
95	*C. trachomatis*	C
96	*C. trachomatis* + *C. psittaci*	C + *C. psittaci*
97	*C. trachomatis* + *C. pecorum*	F + *C. pecorum*
98	*C. trachomatis* + *C. psittaci*	C + *C. psittaci*
99	Negative	Negative
100	*C. trachomatis* + *C. psittaci*	E + *C. psittaci*
101	*C. trachomatis* + *C. psittaci*	C + *C. psittaci*

*ArrayTube (Alere Technologies, Jena, Germany) Negative; *C. trachomatis* positive.
†*ompA* genotypes represent strain type for *C. trachomatis* and/or species.
